# Efficacy and safety at 24 weeks of daily clinical use of tofacitinib in patients with rheumatoid arthritis

**DOI:** 10.1371/journal.pone.0177057

**Published:** 2017-05-04

**Authors:** Naoki Iwamoto, Sosuke Tsuji, Ayuko Takatani, Toshimasa Shimizu, Shoichi Fukui, Masataka Umeda, Ayako Nishino, Yoshiro Horai, Tomohiro Koga, Shin-ya Kawashiri, Toshiyuki Aramaki, Kunihiro Ichinose, Yasuko Hirai, Mami Tamai, Hideki Nakamura, Kaoru Terada, Tomoki Origuchi, Katsumi Eguchi, Yukitaka Ueki, Atsushi Kawakami

**Affiliations:** 1 Department of Immunology and Rheumatology, Unit of Advanced Preventive Medical Sciences, Nagasaki University Graduate School of Biomedical Sciences, Nagasaki, Japan; 2 Department of Rheumatology, Sasebo Chuo Hospital, Sasebo, Japan; 3 Departments of Community Medicine, Unit of Advanced Preventive Medical Sciences, Nagasaki University Graduate School of Biomedical Sciences, Nagasaki, Japan; 4 Department of Physical Therapy, Nagasaki University Graduate School of Biomedical Sciences, Nagasaki, Japan; Keio University, JAPAN

## Abstract

**Objective:**

We evaluated the efficacy and safety of tofacitinib in patients with rheumatoid arthritis (RA) in a real-world setting.

**Methods:**

Seventy consecutive patients, for whom tofacitinib was initiated between November 2013 and May 2016, were enrolled. All patients fulfilled the 2010 ACR/EULAR classification criteria for RA. All patients received 5 mg of tofacitinib twice daily and were followed for 24 weeks. Clinical disease activity indicated by disease activity score (DAS)28-ESR, the simplified disease activity index, and the clinical disease activity index as well as adverse events (AEs) were evaluated. Statistical analysis was performed to determine which baseline variables influenced the efficacy of tofacitinib at 24 weeks.

**Results:**

Fifty-eight patients (82.9%) continued tofacitinib at 24 weeks. Clinical disease activity rapidly and significantly decreased, and this efficacy continued throughout the 24 weeks: i.e., DAS28-ESR decreased from 5.04 ± 1.33 at baseline to 3.83 ± 1.11 at 4 weeks and 3.53 ± 1.17 at 24 weeks (P<0.0001, vs. baseline). 15 AEs including 5 herpes zoster infection occurred during tofacitinib treatment. The efficacy of tofacitinib was not changed in patients without concomitant use of methotrexate (MTX) or patients whose treatment with tocilizumab (TCZ) failed. Multivariable logistic analysis showed that the number of biologic DMARDs (bDMARDs) previously used was independently associated with achievement of DAS-low disease activity.

**Conclusions:**

Our present study suggests that tofacitinib is effective in real-world settings even without concomitant MTX use or after switching from TCZ. Our results also suggest that its efficacy diminishes if started after use of multiple bDMARDs.

## Introduction

Rheumatoid arthritis (RA) is a chronic, systemic disease characterized by inflammation of the synovial joints and associated with significant morbidity and mortality [1]. Biologic DMARDs (bDMARDs) such as tumor necrosis factor inhibitor (TNFi), tocilizumab (TCZ) and abatacept have provided dramatic changes in the management of RA, making remission an achievable goal in many patients [2]. However, not all patients can achieve remission using even with bDMARDs, and some patients are excluded from the benefits of these bDMARDs because of adverse effects, complications and other reasons. Therefore, we need additional therapeutic options for optimal treatment of RA.

Tofacitinib is an oral Janus kinase (JAK) inhibitor which mainly inhibits JAK1 and JAK3 and to a lesser extent, JAK2. Since these JAK families are associated with the cytoplasmic domains of various cytokine receptors such as IL-2, IL-6, IL-7, IL-12, tofacitinib can block signaling for these cytokines. Six phase 3 trials have been conducted to evaluate the efficacy and safety of tofacitinib in patients with RA who responded poorly to other bDMARDs or conventional synthetic DMARDs (csDMARDs), such as TNFi and methotrexate (MTX). Tofacitinib has demonstrated good treatment response in these clinical trials [3–8]. However, up to now, the efficacy and safety of tofacitinib in a real-world setting have been rarely reported because the time period from its approval has been relatively short (Japan, March 2013; US, November 2012) and tofacitinib was not valued as equivalent to other bDMARDs in the 2015 American College of Rheumatology (ACR) guideline for the treatment of RA although the efficacy of tofacitinib appears not to be inferior to that of other bDMARDs[8, 9]. Recently, 2016 European League against Rheumatism (EULAR) recommendations for the management of RA stated that tofacitinib could be considered as first-line molecular-targeted therapy, moreover the route of administration of tofacitinib is oral, so tofacitinib has advantage regarding drug adherence for patients who dislike subcutaneous or intravenous injection [10]. Therefore for best daily clinical practice for management of RA, we need evidence of “real-world” effectiveness and safety of tofacitinib. Here, we evaluated the efficacy and safety of tofacitinib in patients with RA in a real-world setting.

## Patients and method

### Patients

All patients were registered at the Department of Immunology and Rheumatology, Nagasaki University Graduate School of Biomedical Sciences and Sasebo Chuo Hospital, and received their follow-up there. A total of 70 consecutive patients, in whom tofacitinib was initiated between November 2013 and May 2016, were enrolled. All patients fulfilled the 2010 ACR/EULAR classification criteria for RA. The patients gave their informed consent to be subjected to the protocol, which was approved by the Institutional Review Board of Nagasaki University (IRB approval number: 11032819–2) and Sasebo Chuo Hospital (IRB approval number: 2011–4). Demographic data recorded at the initiation of tofacitinib including age, sex, disease duration, rheumatoid factor (RF), anti-citrullinated protein antibodies, history of previous DMARDs, concomitant medications. All patients received 5 mg of tofacitinib twice daily. Concomitant csDMARD therapy was not changed during the 24-week observation period. The Japan College of Rheumatology (JCR) has a guideline regarding the use of tofacitinib which states that tofacitinib is recommended for RA patients who are refractory to MTX > 8 mg treatment for at least 3 months at present or past (http://www.ryumachi-jp.com/info/guideline_tofacitinib.html). Sixty-seven of the 70 patients included in this study fit that description. The remaining 3 patients were MTX-naïve but had been refractory to conventional therapy including other bDMARDs.

### The evaluations for efficacy and safety

Clinical disease activity as indicated by the disease activity score (DAS)28-ESR, simplified disease activity index (SDAI), and clinical disease activity index (CDAI) were evaluated at 0, 4, 8, 12, 16, 20, and 24 weeks after the initiation of tofacitinib treatment. Disease activity was categorized as follows: DAS 28-ESR remission (DAS28-ESR<2.6), CDAI remission (CDAI<2.8), SDAI remission (SDAI<3.3), DAS28-ESR low disease activity (LDA) (2.6≤DAS28-ESR≤3.2), CDAI LDA (2.8≤CDAI≤10), SDAI LDA (3.3≤SDAI≤11), DAS28-ESR moderate disease activity (MDA) (3.2<DAS28-ESR≤5.1), CDAI MDA (10<CDAI≤22), SDAI LDA (11<SDAI≤26) and DAS28-ESR high disease activity (HDA) (DAS28-ESR>5.1), CDAI HDA (CDAI>22), SDAI HDA (SDAI >26). These criteria were established in a previous report [11, 12]. Functional disability was assessed using Health Assessment Questionnaire disability index (HAQ-DI). Adverse events (AEs) until 24 weeks were also analyzed.

### Statistical analysis

GraphPad prism software (GraphPad Software, San Diego, CA) and JMP Statistical Software (SAS Institute, Cary, NC) were used for statistical analysis. The distribution of baseline variables and proportion of disease activity in different patient subgroups were examined by Mann-Whitney U test and chi-square test. For patients who withdrew before week 24 and in cases of missing data, the last observation carried forward (LOCF) method was employed. The Wilcoxon signed rank test was used to detect significant differences in disease activity. The survival rate of tofacitinib was assessed using the Kaplan-Meier method and the log-rank test was used to compare survival rates across treatment groups. Univariate and multivariable ordinal logistic regression analyses were used to determine the predictive factor of clinical responses. Variables with p< 0.25 in the univariate logistic regression analyses were entered in the multivariate logistic regression analysis. P-values less than 0.05 were considered to indicate statistical significance.

## Results

### Baseline characteristics of the patients and drug survival at 24 weeks

A total of 70 patients were enrolled in this study. Baseline demographic characteristics are illustrated in Table 1. The mean age of patients was 64.2 ± 11.5 years, and the majority of the subjects were women (84.3%). The mean duration of disease was 16.4 ± 10.0 years. The percentage of patients who used MTX concomitantly with tofacitinib was 68.6% while 52.9% of patients used oral steroid concomitantly. Most of the patients had experienced bDMARDs (1 or 2 bDMARDs before tofacitinib, 24 patients; ≥3 bDMARDs before tofacitinib, 24 patients), and 22 patients were bDMARDs-naïve. At baseline, the mean DAS28-ESR, SDAI, and CDAI values were 5.04 ± 1.33, 22.25 ± 14.17, and 21.66 ± 13.60, respectively. At the end of the 24-week follow-up period, fifty-eight patients (82.9%) were still taking tofacitinib. Twelve cases discontinued the treatment: 7 due to lack of efficacy, 4 due to an AEs (pneumonia, sepsis, nausea, parotid cancer), and remaining 1 due to patient’s choice.

**Table 1:** Clinical characteristics of the study population.

Female, n (%)	59 (84.3)
Age (years)	64.2 ± 11.5
Duration of RA (year)	16.4 ± 10.0
Steinbrocker stage scores (I/II/III/IV %)	(12.9/15.7/12.9/58.5)
Steinbrocker class scores (1/2/3/4%)	(12.9/82.8/4.3/0.0)
No prior use of biologic DMARDs, n (%)	22 (31.4)
Prior use of 1 biologic DMARDs, n (%)	6 (8.6)
Prior use of 2 biologic DMARDs, n (%)	18 (25.7)
Prior use of 3 or more biologic DMARDs, n (%)	24 (34.3)
Concomitant MTX use, n (%)	48 (68.6)
MTX dose (mg/week)	8.5 ± 1.9
Concomitant oral steroid use, n (%)	37 (52.9)
Oral steroid dose (mg/day)	4.6 ± 3.0
ACPA positive, n (%)	54 (77.1)
RF positive, n (%)	53 (75.7)
Tender 28-joint count, (median [IQR])	6.5 [3.0–10.3]
Swollen 28-joint count, (median [IQR])	2.0 [1.0–4.0]
HAQ-DI, (median [IQR])	0.3 [0–1.0]
ESR(mm/h), (median [IQR])	41.0 [15.0–63.0]
CRP(mg/dl), (median [IQR])	0.37 [0.04–2.43]
PGA, VAS 0–100 mm,(median [IQR])	30 [20–50]
PtGA, VAS 0–100 mm,(median [IQR])	38 [20–52]
DAS28-ESR	5.04 ± 1.33
SDAI	22.25 ± 14.17
CDAI	21.66 ± 13.60

Data are mean ± standard deviation (SD) unless otherwise indicated.

*RA* rheumatoid arthritis, *DMARDs* disease-modifying antirheumatic drugs, *MTX* methotrexate, *ACPA* anti-citrullinated protein antibodies, *RF* rheumatoid factor, *ESR* erythrocyte sedimentation rate, *CRP* C-reactive protein, *PGA* physician global assessment of disease activity, *VAS* visual analogue scale, *PtGA* patient global assessment of disease activity, *DAS* disease activity score, *HAQ-DI* health assessment questionnaire disability index, *SDAI* simplified disease activity index, *CDAI* clinical disease activity index, *IQR* interquartile range

### Overall efficacy and safety of tofacitinib treatment

**Fig 1:**
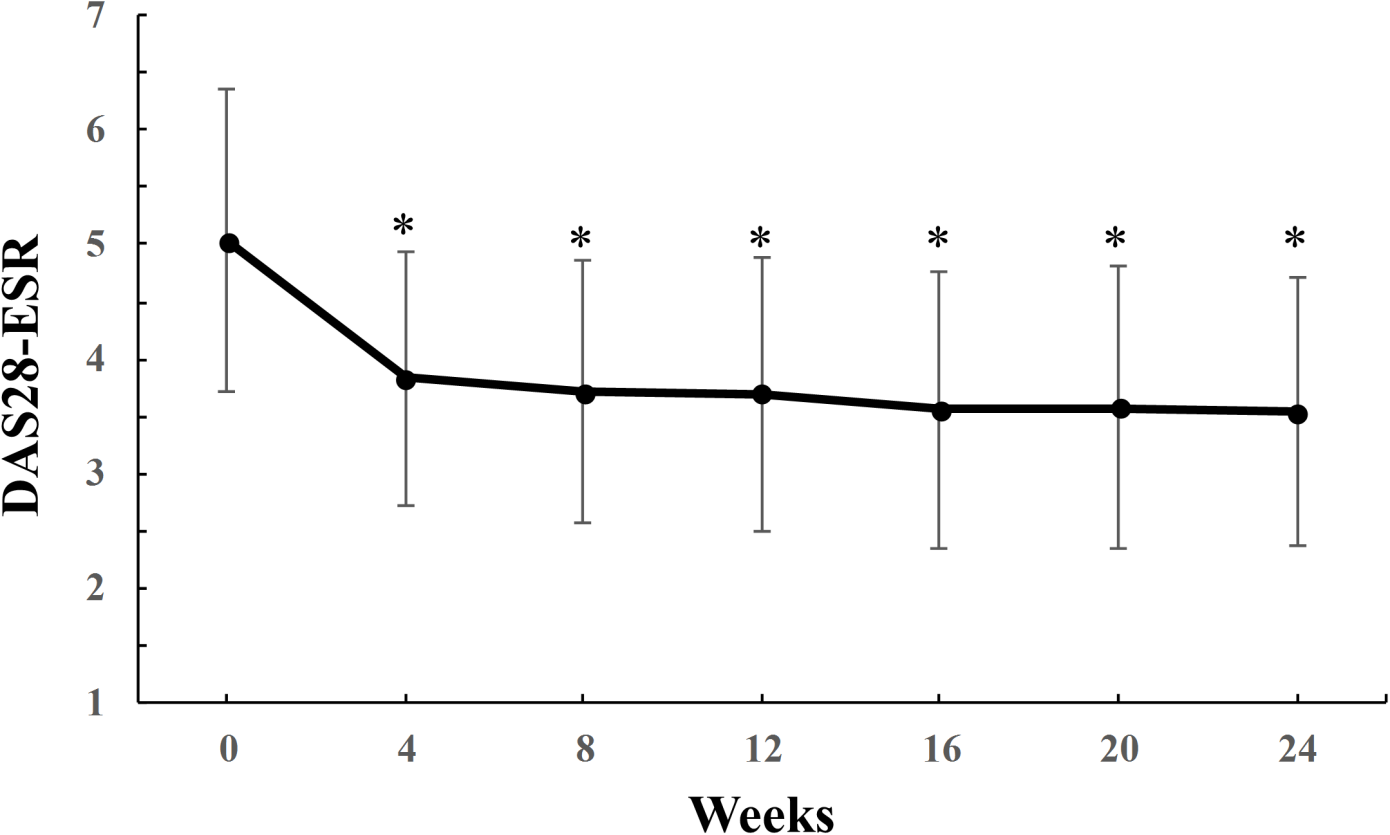
Time course of disease activity scores over 24 weeks of tofacitinib treatment. Data were analyzed by the LOCF method. Points and bars represent means and standard deviations, respectively. *p < 0.0001 versus baseline by the Wilcoxon signed rank test. *LOCF* last observation carried forward, *ESR* erythrocyte sedimentation rate, *DAS* disease activity score.

**Fig 2:**
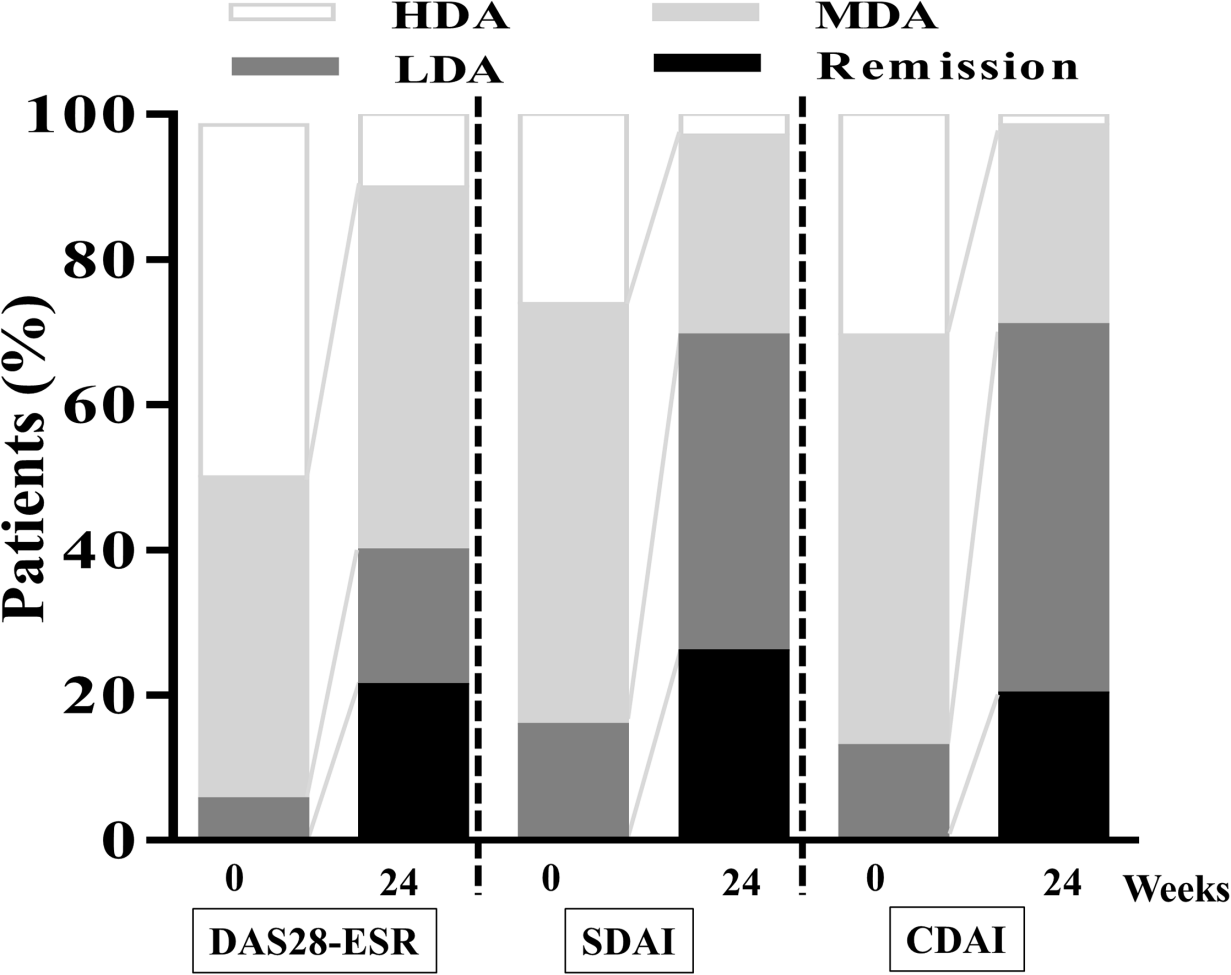
The proportion of disease activity at baseline and 24 weeks after initiation of tofacitinib treatment. Disease activity was categorized as follows: DAS 28-ESR <2.6(remission), 2.6-≤3.2(LDA), 3.2-≤5.1(MDA), 5.1<(HDA); SDAI<3.3(remission), 3.3-<11(LDA), 11-≦26 (MDA), 26<(HDA); CDAI<2.8(remission), 2.8-<10(LDA), 10-≦22 (MDA), 22<(HDA); *ESR* erythrocyte sedimentation rate, *DAS* disease activity score, *SDAI* simplified disease activity index, *CDAI* clinical disease activity index, *LDA* low disease activity, *MDA* moderate disease activity, *HDA* high disease activity.

As shown in Fig 1, the mean DAS28-ESR score rapidly and significantly decreased from 5.04 ± 1.33 at baseline to 3.83 ± 1.11 at 4 weeks (P<0.0001), and this efficacy of tofacitinib continued throughout the 24 weeks: the DAS28-ESR score was 3.69 ± 1.19 at 12 weeks, and 3.53 ± 1.17 at 24 weeks. The proportion of disease activity defined by DAS28-ESR, SDAI, and CDAI are shown in Fig 2. The proportion of patients who achieved remission significantly increased from baseline to 24 weeks for each clinical index (DAS28-ESR: 0% to 21.4%, SDAI: 0% to 26.1%, CDAI: 0% to 20.3%). As well as remission, the proportion of patients who achieved less than LDA significantly increased during 24 weeks (DAS28-ESR: 5.7% to 40.0%, SDAI: 15.9% to 69.6%, CDAI: 13.0% to 71.0%). HAQ-DI values decreased from 0.3 [0–1.0] at baseline to 0.1 [0–0.88] at 24 weeks (median [interquartile range], P<0.05). Fourteen patients (20.0%) experienced one or two AEs during 24 weeks. All AEs are reported in Table 2. As expected, the most common AEs were infection (15.7%), especially herpes zoster (five patients). The herpes zoster infections occurred at 10–22 weeks after the initiation of tofacitinib, and all five patients recovered within 3 weeks and then restarted tofacitinib treatment. Among patients who experienced AEs, 4 patient discontinued tofacitinib treatment.

**Table 2:** Adverse events.

	Number of events
Infection	
Herpes zoster	5
Pneumonia	3
Upper respiratory infection	2
Sepsis	1
Gastrointestinal disorder	
Nausea	1
Diarrhea	1
Neoplasm	
Parotid cancer	1
Skin disorders	
Rash	1

### Tofacitinib was effective in patients without concomitant use of MTX and patients who failed treatment with TCZ

**Fig 3:**
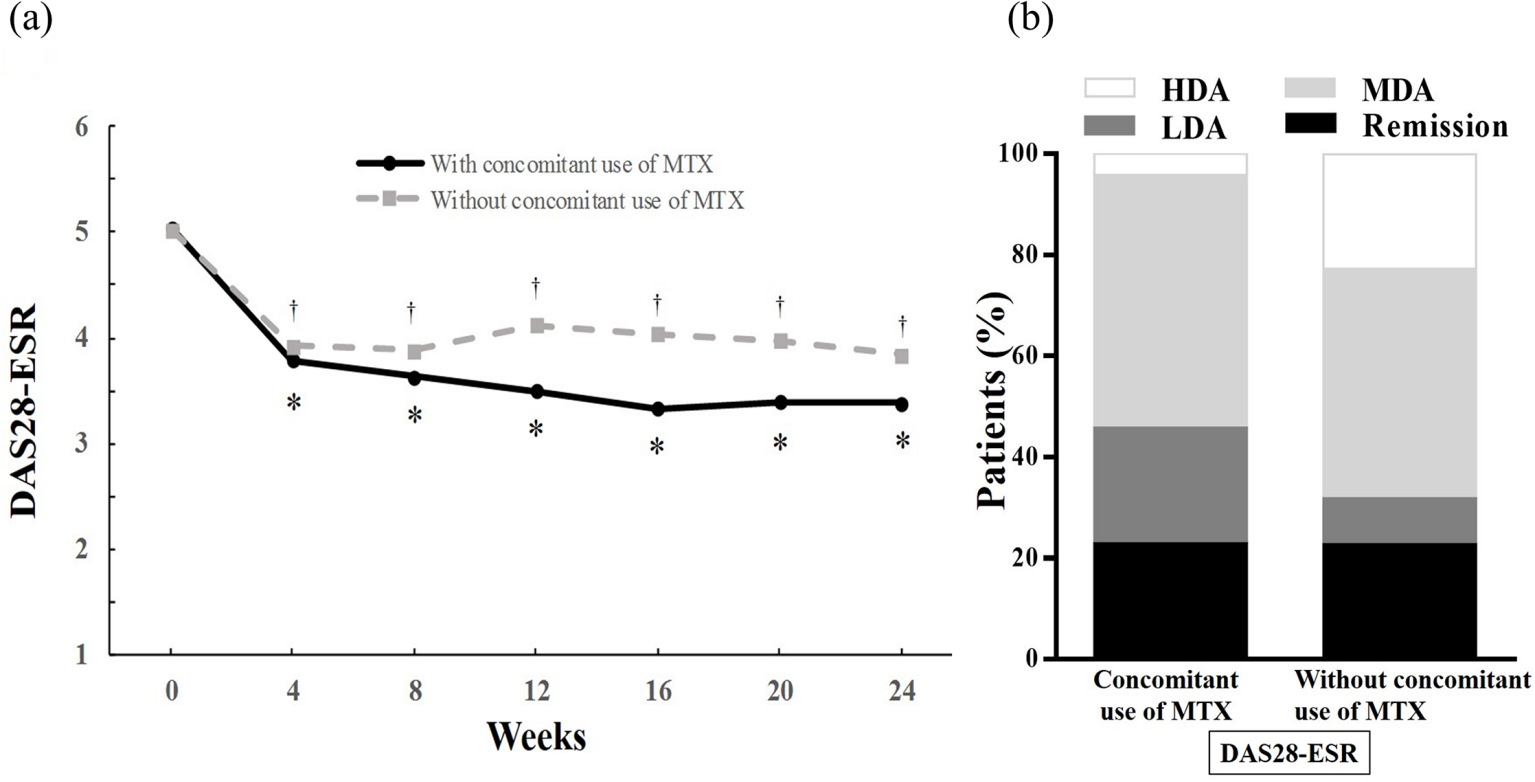
**(a) Time course of the disease activity score over 24 weeks following the initiation of tofacitinib treatment with or without concomitant use of MTX.** Data were analyzed by the LOCF method. Points represent means. * p < 0.0001 versus baseline by the Wilcoxon signed rank test. † p < 0.05 versus baseline by the Wilcoxon signed rank test. **(b) The proportion of disease activity at 24 weeks after initiation of tofacitinib treatment with or without concomitant use of MTX.** Disease activity was categorized as follows. DAS 28-ESR <2.6(remission), 2.6-≤3.2(LDA), 3.2-≤5.1(MDA), 5.1<(HDA). *MTX* methotrexate, *LOCF* last observation carried forward, *ESR* erythrocyte sedimentation rate, *DAS* disease activity score, *LDA* low disease activity, *MDA* moderate disease activity, *HDA* high disease activity.

A comparison of subgroups divided by concomitant use of MTX or not, and after switching from TCZ or not, is provided in Table 3. In switching from TCZ group, the disease activity, concomitant oral steroid use and ESR were higher as compared with non-switching from TCZ group. Whereas, Most of the baseline variables including disease activity were equivalent among subgroups divided by concomitant use of MTX except RF positivity. There was no statistical significance in the survival rate of tofacitinib at 24 weeks between with and without concomitant use of MTX (85.4% and 77.3%, respectively, P = 0.37, survival plot is available upon request from the corresponding author). Likewise, a decrement of DAS28-ESR was found in RA patients without concomitant use of MTX as well as in those with MTX (Fig 3A). The mean DAS28-ESR score decreased from 5.04 ± 1.31 at baseline to 3.39 ± 1.15 at 24 weeks with concomitant use of MTX, from 5.02 ± 1.41 at baseline to 3.84 ± 1.35 at 24 weeks without concomitant use of MTX. The proportion of disease activity at 24 weeks defined by DAS28-ESR is shown in Fig 3B. There were no significant differences in the above indices between those with and without MTX.

**Table 3:** Comparison of baseline characteristics in patients with different backgrounds.

	Concomitant use of MTX	No concomitant use of MTX	Switching from TCZ	No switching from TCZ
Number of patients	48	22	24	46
Female, n (%)	38 (79.2)	21 (95.5)	20 (83.3)	39 (84.8)
Age (years)	62.9 ± 10.6	67.0 ± 13.0	63.8 ± 11.6	64.4 ± 11.6
Duration of RA (year)	14.9 ± 8.6	19.8 ± 12.3	17.7 ± 7.2	15.8 ± 11.2
Concomitant MTX use, n (%)			14 (58.3)	35 (58.3)
MTX dose (mg/week)			8.4 ± 1.6	8.5 ± 2.1
Concomitant oral steroid use, n (%)	26 (54.2)	11 (50.0)	7 (29.2)	30 (65.2)*
Oral steroid dose (mg/day)	3.85 ± 2.5	6.2 ± 3.5	4.9 ± 3.8	4.5 ± 2.8
ACPA positive, n (%)	37 (77.1)	17 (77.3)	19 (79.2)	35 (76.1)
RF positive, n (%)	42 (87.5)	11 (50.0) **	15 (62.5)	38 (82.6)
Tender 28-joint count, (median [IQR])	7.0 [3.0–10.0]	6.0 [3.8–12.3]	6.0 [3.3–10.8]	7.0 [3.0–10.5]
Swollen 28-joint count, (median [IQR])	2.0 [1.0–4.0]	2.0 [2.0–4.0]	2.0 [2.0–3.0]	2.0 [1.0–6.0]
HAQ-DI, (median [IQR])	0.28 [0–0.8]	0.63 [0.1–1.5]	0.5 [0.1–1.0]	0.25 [0–1.0]
ESR(mm/h), (median [IQR])	40.0 [14.0–64.0]	47.5 [15.0–58.8]	18.5 [9.3–46.0]	49.0 [23.0–70]*
CRP(mg/dl), (median [IQR])	0.37 [0.03–1.99]	0.34 [0.05–2.74]	0.16 [0.01–1.07]	0.44 [0.06–2.74]
PGA,VAS 0–100 mm, (median [IQR])	30 [20–50]	30 [20–53]	30 [20–49]	30 [15–50]
PtGA,VAS 0-100mm, (median [IQR])	38 [20–57]	38 [20–50]	30 [20–49]	40 [20–60]
DAS28-ESR	5.04 ± 1.30	5.02 ± 1.41	4.33 ± 1.10	5.40 ± 1.30*
SDAI	22.01 ± 14.16	18.51 ± 9.03	17.76 ± 8.56	24.65 ± 15.97
CDAI	21.44 ± 13.48	17.65 ± 8.71	16.98 ± 8.61	24.16 ± 15.12*

Data are mean ± standard deviation (SD) unless otherwise indicated.

*RA* rheumatoid arthritis, *MTX* methotrexate, *TCZ* tocilizumab, *CRP* C-reactive protein, *ESR* erythrocyte sedimentation rate, *PGA* physician global assessment of disease activity, *VAS* visual analogue scale, *PtGA* patient global assessment of disease activity, *DAS* disease activity score, *HAQ-DI* health assessment questionnaire disability index, *SDAI* simplified disease activity index, *CDAI* clinical disease activity index, *IQR* interquartile range

* P<0.05 versus switching from TCZ group

** P<0.05 versus concomitant use of MTX group.

**Fig 4:**
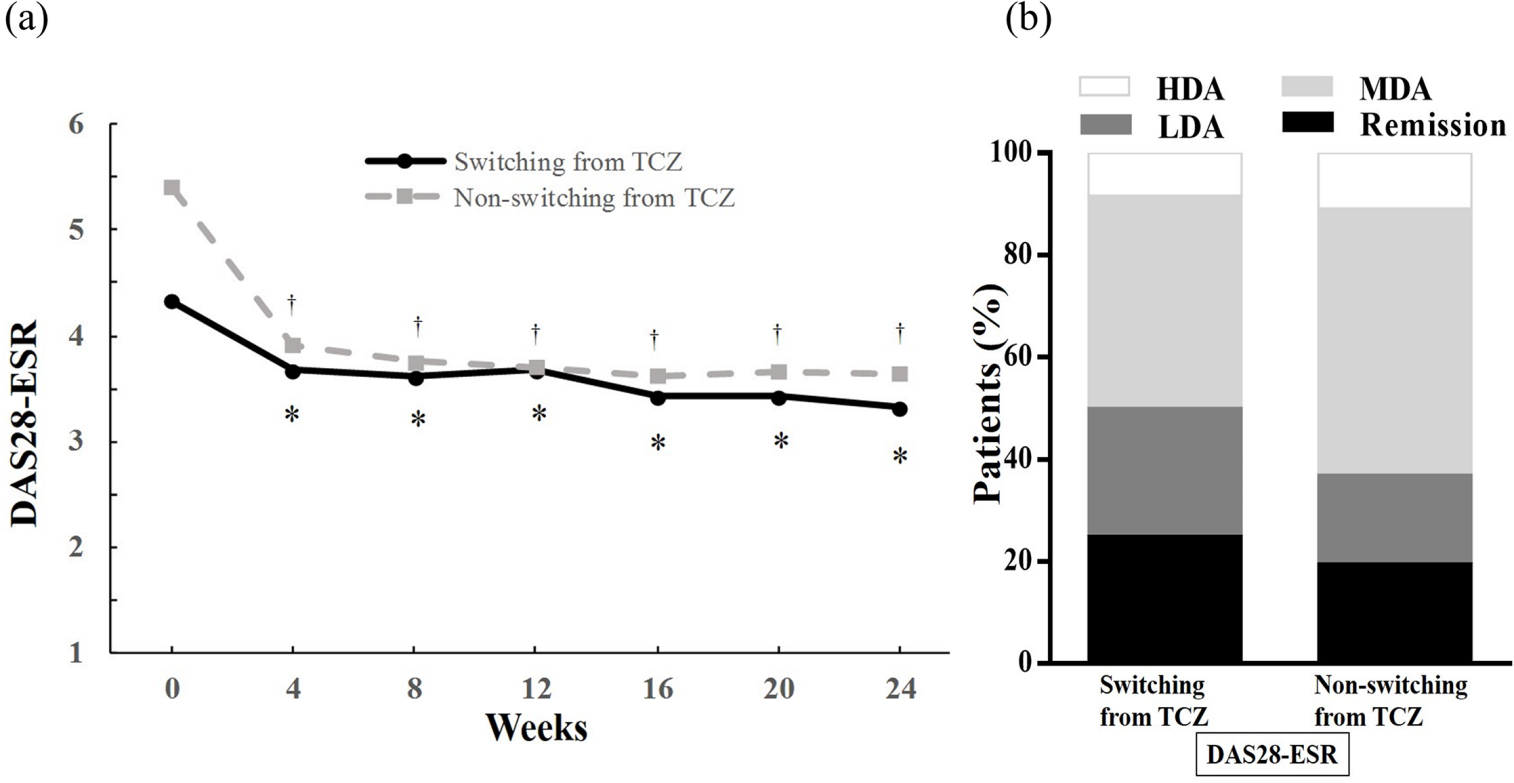
**(a) Time course of the disease activity score over 24 weeks following the initiation of tofacitinib treatment after switching from TCZ or not.** Data were analyzed by the LOCF method. Points represent means. * p < 0.05 versus baseline by the Wilcoxon signed rank test. † p < 0.0001 versus baseline by the Wilcoxon signed rank test. **(b) The proportion of diseased activity at 24 weeks after initiation of tofacitinib treatment after switching from TCZ or not.** Disease activity was categorized as follows. DAS 28-ESR <2.6(remission), 2.6-≤3.2(LDA), 3.2-≤5.1(MDA), 5.1<(HDA). *LOCF* last observation carried forward, *TCZ* tocilizumab, *ESR* erythrocyte sedimentation rat, *DAS* disease activity score, *LDA* low disease activity, *MDA* moderate disease activity, *HDA* high disease activity.

Tofacitinib suppresses multiple cytokines including IL-6 through inhibition of JAK signaling. Therefore, we next analyzed the efficacy of tofacitinib in patients with failed treatment by TCZ, an IL-6 inhibitor. In this study, TCZ was switched to tofacitinib in 24 patients because of lack of efficacy. DAS28-ESR improved significantly from 4.33 ± 1.10 at baseline to 3.32 ± 0.98 at 24 weeks in these 24 patients (Fig 4A) and DAS28-ESR remission at 24 weeks was attained by 25.0% (Fig 4B). Although a direct comparison is not possible because of the differences in baseline characteristics, tofacitinib seemed less effective in switching from TCZ group compared to the ‘no switching from TCZ’ group. However, there was no significant difference in efficacy between these two groups. Moreover, compared with switching from TNF inhibitor group (n = 10), the efficacy of tofacitinib was comparable among both groups (the mean Δ values in DAS28-ESR between baseline and 24 weeks after the initiation of tofacitinib was -1.01 in switching from TCZ group, -0.88 in switching from TNF inhibitor group, respectively, P = 0.88). Taken together, these data indicate that tofacitinib was effective in the patients who showed inadequate response to IL-6 inhibitor.

### Factors predicting achievement of good clinical response

The predictors of clinical response throughout the study period in univariate logistic analysis are shown in Table 4. Univariate analysis showed that the number of previous use of bDMARDs, DAS-HDA at baseline and bDMARDs naïve were associated with achievement of DAS-LDA at 24 weeks. Multivariable logistic analysis showed that the number of bDMARDs previously used was independently and inversely associated with achievement of DAS-LDA.

**Table 4:** Multivariate regression model to estimate the factors associated the achievement of LDA at 24 weeks.

Variables	Univariate model		Multivariable model	
	OR (95% CI)	p-value	OR (95% CI)	p-value
Age (per 1-year increase)	1.018 (0.976–1.063)	0.42		
Disease duration (per 1-year increase)	0.976 (0.0.924–1.025)	0.34		
Concomitant MTX use (yes/no)	1.667 (0.589–5.050)	0.34		
Concomitant oral steroid use (yes/no)	0.650 (0.245–1.697)	0.38		
No previous use of bDMARDs (yes/no)	2.071 (0.734–5.957)	0.17	1.961 (0.306–13.91)	0.48
Number of previous use of bDMARDs (per drug)	0.671 (0.465–0.929)	0.02*	0.551 (0.269–0.994)	0.047*
DAS-HDA at baseline (yes/no)	0.535 (0.198–1.400)	0.20	0.561 (0.193–1.570)	0.27
ESR (above the normal range) (yes/no)	0.703 (0.231–2.156)	0.53		
ACPA positive (yes/no)	0.588 (0.188–1.831)	0.37		
RF positive (yes/no)	0.938 (0.310–2.944)	0.91		

*OR* odds raio, *95% CI* 95% confidence interval, *MTX* methotrexate, *bDMARDs* biologic disease-modifying antirheumatic drugs, *HDA* high disease activity, *ESR* erythrocyte sedimentation rate, *ACPA* anti-citrullinated protein antibodies, *RF* rheumatoid factor.

* P<0.05

## Discussion

We evaluated the efficacy and safety of tofacitinib in a “real-world” setting. The results of this study showed the benefit of tofacitinib treatment in patients with variable backgrounds. Generally, as compared with randomized controlled trials (RCTs), an observational study like this one includes patients who have various characteristics and treatment histories, as found in daily practice. Therefore, such clinical observational studies sometimes provide very useful information for clinicians. Our study showed that tofacitinib is effective even in patients without concomitant use of MTX and patients who were switched from TCZ because of lack of efficacy. We compared the survival rate of tofacitinib between those with concomitant MTX and without, and found no statistical difference between the two groups. Regarding efficacy, tofacitinib was effective even in the group without concomitant MTX; 22.7% of patients in this group achieved DAS28-ESR remission at 24 weeks. This result is strongly consistent with the result from RCTs. Phase 3 trials called ORAL solo which evaluated the efficacy of tofacitinib monotherapy demonstrated a better ACR20/50/70 response rate at 3 months (59.8%, 31.1%, 15.4% respectively) than with the placebo (26.7%, 12.5%, 5.8%, respectively)[4]. Recent meta-analysis using a Bayesian method showed that tofacitinib with concomitant use of MTX was superior to tofacitinib without concomitant use of MTX; however, we should interpret these results with caution because there were no direct comparison among these two groups[13]. In our study, although concomitant use of MTX did not cause a statistical difference in the efficacy, tofacitinib with concomitant use of MTX appeared to be more effective than that without concomitant MTX (the mean Δ values in DAS28-ESR between baseline and 24 weeks after the initiation of tofacitinib was -1.73 with MTX, -1.18 without MTX, respectively). There is growing interest in switching strategy for RA patients who have failed to treated bDMARDs. Several studies have reported that if inadequate responses were provided by more than one TNFi, switching to a non-TNFi bDMARD may provide a better clinical outcome than switching to a third TNFi [14–17]. Furthermore, a recent report showed that switching to rituximab after discontinuation of an initial TNFi was associated with improved clinical effectiveness as compared with switching to a second TNFi[18]. Considering these results, tofacitinib may be effective in patients who have shown inadequate response to other bDMARDs. Because tofacitinib can inhibit multiple cytokines besides IL-6, tofacitinib may control RA disease activity by inhibiting other pathways of RA pathology over the TNF-IL-6 axis in patients with TNFi/TCZ treatment failure. Indeed, our study demonstrated that switching to tofacitinib from TCZ led to improvement in disease activity at 24 weeks. Although the number of patients in this study was small, we analyzed the various factors associated with the response to tofacitinib. Multivariate analysis revealed that number of bDMARDs previously used was associated with achievement of DAS-LDA. bDMARDs-naïve patients have shown better treatment response as compared with bDMARDs-experienced patients in many studies of bDMARDs[19]. Our result may reflect that treatment with tofacitinib has a similar tendency with regard to prior use of bDMARDs: namely, that use of fewer bDMARDs prior to tofacitinib predicts a better response to treatment. Such data are very important to management of RA using tofacitinib. We should consider use of tofacitinib before multiple bDMARDs failure, although treatment by tofacitinib as first molecular-targeted therapy should be approached cautiously because there are few long-term experience as compared with other bDMARDs.

AEs occurred in 20.0% of patients. As with other RCTs, infection was the most frequent AE to tofacitinib seen in our study. Among infection types, herpes zoster was the most frequent. As compared with published results about treatment with other bDMARDs, the incidence rate of herpes zoster elevated in clinical trials and long-term extension studies [20].Although the mechanism by which tofacitinib cause herpes zoster still remains unclear, CD4 T cell function and regulation of interferons during viral infections are both suspected to be factors. *In vitro*, tofacitinib diminishes CD4 T-cell function[21], and innate antiviral defenses through interferon signaling has been found to be dependent on JAK 1 receptors[22]. With respect to malignancy, tofacitinib has been concerned with risk of malignancy because tofacitinib is an immunomodulatory drug with new mechanisms of action, and NK cell numbers do decline during tofacitinib treatment [23, 24]. However, a recent meta-analysis revealed that the incidence of malignancy during tofacitinib treatment was not higher than that with other treatment[25]. More studies are needed to investigate the risk of malignancy in long-term extension studies and studies in real-world settings.

Several limitation of this study must be mentioned. The number of patients was small and the long-term efficacy and safety of tofacitinib was not explored. Although this small number analysis is meaningful because the result from this study was consistent with previous large RCTs even in a real-world setting, longer observations and a larger number of observations in real-world settings are needed for adaptive use of tofacitinib in daily clinical practice. Moreover, the statistical reliability of multivariate analysis that we performed in this study was not so strong because of the analysis of small numbers; analysis of larger numbers is needed to validate our results. In addition, regarding our comparison of efficacy concerning the concomitant use of MTX and switching from TCZ, the patient backgrounds including disease activity of each group were quite variable, and this difference might affect the efficacy of tofacitinib. We need to test our present findings in background-matched patients. Generally, patients prefer oral drugs as compared with subcutaneous injection or intravenous injection therapy. For patients who have used other bDMARDs previously, tofacitinib relieves the pain or discomfort caused by subcutaneous injection or intravenous injection. We did not measure patient satisfaction with tofacitinib treatment in this study. Further analysis from the viewpoint of patient satisfaction is also needed.

In conclusion, our present study suggests that tofacitinib treatment is effective in real-world settings even without concomitant use of MTX, or after switching from TCZ. The incidence of AEs was comparable with previous RCTs even in a real-world setting. Tofacitinib treatment is a useful choice in patients with inadequate response to bDMARDs or with MTX intolerance.
